# Fluorescein- and EGFR-Antibody Conjugated Silica Nanoparticles for Enhancement of Real-time Tumor Border Definition Using Confocal Laser Endomicroscopy in Squamous Cell Carcinoma of the Head and Neck

**DOI:** 10.3390/nano9101378

**Published:** 2019-09-26

**Authors:** Anna Watermann, Rita Gieringer, Anna-Maria Bauer, Sven Kurch, Ralf Kiesslich, Wolfgang Tremel, Jan Gosepath, Juergen Brieger

**Affiliations:** 1 Molecular Tumorbiology, Department of Otorhinolaryngology, Head and Neck Surgery, University Medical Center of the Johannes Gutenberg University Mainz, 55131 Mainz, Germany; awaterma@uni-mainz.de (A.W.);; 2 Institute of Inorganic Chemistry and Analytical Chemistry, Johannes Gutenberg University, 55128 Mainz, Germany; 3 HSK Klinik für Innere Medizin II: Gastroenterologie, Hepatologie und Endokrinologie, 65199 Wiesbaden, Germany; 4 HSK Klinik für Hals-, Nasen- und Ohrenheilkunde, Kopf- und Hals-Chirugie, 65199 Wiesbaden, Germany

**Keywords:** contrast agent, silica nanoparticles, EGFR

## Abstract

Intraoperative definition of tumor free resection margins in head and neck cancer is challenging. In the current proof-of-principle study we evaluated a novel silica nanoparticle-based agent for its potential use as contrast enhancer. We synthesized silica nanoparticles with an average size of 45 nm and modified these particles with the fluorescence stain fluorescein isocyanate (FITC) for particle detection and with epidermal growth factor receptor (EGFR)-targeting antibodies for enhanced tumor specificity. The nanoparticles exhibited good biocompatibility and could be detected in vitro and in vivo by confocal laser scanning microscopy. Additionally, we show in an ex vivo setting that these modified nanoparticles specifically bind to tumor samples and could be detected using a handheld confocal fluorescence endomicroscope. From a clinical point of view, we believe that this method could be used for tumor border contrast enhancement and for better intraoperative definition of R-0 tumor resection.

## 1. Introduction

Tumors of the upper aero digestive tract comprise malignant diseases of the head and neck, the nose and nasal cavities and the larynx. Typically, in case of lesions suspicious of squamous cell carcinoma, an endoscopic in vivo evaluation of the mucosa is performed. In the event of a surgical procedure, the tumor border definition is the most challenging, i.e., the discrimination of malignant and pre-malignant epithelial areas from healthy, non-affected squamous epithelia and of invasive cells [1]. The current gold standard for definitive evaluation of pathological epithelial changes is biopsy followed by histopathologic examination. To this end, the surgeon takes multiple samples of suspicious epithelia after visual macroscopical appraisal. So-called secondary resection material from macroscopically unsuspicious tumor border is collected for examination in case of previous tumor resection and to document complete removal of the lesion. Another diagnostic tool is the rapid incision examination mainly used during surgery for quick analysis of tumor free resection margins. The main disadvantage of this application is the time needed until the result is available. When the suspicious tissue is found to be malignant, an immediate follow-up surgery will be performed.

Endomicroscopy enables the in vivo microscopic analysis of the mucosa up to a depth of 250 µm in 1100× magnification during ongoing endoscopic examination of the patient. For this purpose, a miniaturized confocal microscope has been integrated into the distal end of a conventional flexible endoscope. This method has been established by us for colonoscopy before [2]. Using this so-called endomicroscope, subcellular details could be visualized after application of fluorescence stains. This technique is a novelty in endoscopic imaging because it enables real time in vivo histology. The high clinical and scientific potential of this technique has been verified by us in several clinical and translational gastrointestinal studies [2,3,4,5].

An extension of the indication for the use of this endomicroscope to less accessible locations such as the larynx or hypopharynx was enabled by the development of a prototype of a rigid hand held confocal endomicroscope [6]. We assessed the value of this microscopy technique for the diagnostic evaluation of 15 patients with squamous cell carcinoma of the upper aerodigestive tract and were able to show that differentiation of normal, healthy epithelia from malignant squamous cell carcinoma by using confocal laser endomicroscopy is feasible. Comparison of the real time results with conventional histology confirmed the validity of the method [6]. However, despite these promising experiences, the need of further contrast enhancement of imaging became evident.

Nanoparticles (NPs) are a novel class of drug carriers with specific advantageous properties making them a promising tool for contrast enhancement. E.g., nanoparticles accumulate passively in tumors due to the enhanced permeability and retention (EPR) effect [7]. Second, depending on the NP modifications, e.g., with tumor specific ligands or fluorophores, detection or enhancement of specificity is possible. Silica nanoparticles (SiO_2_-NPs)—solid, as well as mesoporous ones—are currently under evaluation as drug delivery systems [8]. Apart from the above-mentioned general advantages of NPs, SiO_2_-NPs have several additional favorable properties: their synthesis is simple and cheap, final degradation solely delivers the trace element silicon (Si) and particles are non-toxic. Additionally, silica is classified as “generally recognized as safe” (GRAS) by the FDA [9]. 

The epidermal growth factor receptor (EGFR) is up-regulated on most squamous cell carcinoma cells and dysplastic precursor lesions and has been identified as a drug target for immunotherapy [10,11]. Hence, the EGF receptor is an adequate molecule for potential enhancement of tumor specific nanoparticle targeting. The fluorophore fluorescein (FITC) is established in human use for contrast enhancement [1,2,12]. However, the accuracy of fluorescein sodium administered intravenously did not meet the expectations when applied for imaging of oral and oropharyngeal mucosa in humans [1]. We therefore decided to synthesize fluorescein labeled SiO_2_-NPs tagged with anti-EGF-receptor-antibodies (EGFR-SiO_2_-NPs) and evaluated this novel contrast agent for tumor specific targeting in an ex vivo model system. 

We analyzed in this proof-of-principle study a novel method for enhanced, reliable and easy discrimination of head and neck cancer tissue and normal, healthy mucosa. The specific targeting of the EGFR typically expressed at high levels on squamous cell carcinoma by using biocompatible anti-EGFR-antibody and FITC tagged silica nanoparticles is a promising approach for contrast enhancement and could foster the use of confocal endomicroscopy in head and neck oncology.

## 2. Materials and Methods 

**Nanoparticle synthesis:** SiO_2_ nanoparticles (average diameter: 45 nm) were synthesized according to the procedure reported by Stöber et al. with slight modifications [13]. In brief, 35 mL of ethanol (99.8%, Carl Roth, Karlsruhe, Germany), 5 mL of double-distilled water and 1 mL of ammonium hydroxide (25% aqueous solution, Sigma-Aldrich, St. Louis, MO, USA) were mixed and heated to 70 °C. Subsequently, tetraethyl orthosilicate (>99%, Sigma-Aldrich, St. Louis, MO, USA) was added at once, and the reaction mixture was stirred for one hour while being kept at 70 °C. The reaction was stopped by centrifugation at 9000 rpm for 10 min, and the SiO_2_-NPs were washed repeatedly with ethanol and finally, were stored in ethanol at 4 °C.

**Figure 1:**
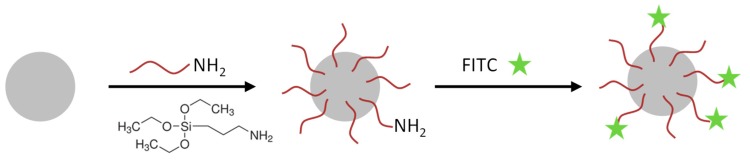
Cartoon of the functionalization procedure of SiO_2_ nanoparticles. SiO_2_-NPs were functionalized with APTES to obtain reactive amino groups. In the next step, 5(6)-carboxyfluorescein N-hydroxysuccinimide ester (FITC) was conjugated to some amino groups (FITC-SiO_2_-NPs).

**Figure 2:**
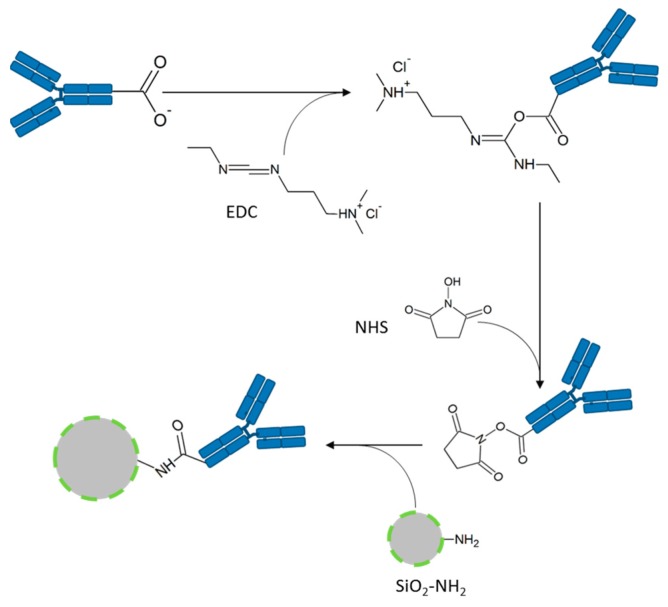
EGFR-antibody conjugation to FITC-SiO_2_-NPs. First, the EGFR-antibodies were activated with 1-ethyl-3-(-3-dimethylaminopropyl) carbodiimide hydrochloride (EDC) and N-hydroxysuccinimide (NHS). In the second step, the semi-stable NHS ester formed an amide bond with the amine-functionalized FITC-SiO_2_-NPs. Hence, EGFR-antibodies were covalently bound to FITC-SiO_2_-NPs resulting in EGFR-FITC-SiO_2_-NPs.

**Nanoparticle functionalization and EGFR-antibody coupling:** The SiO_2_-NPs were functionalized with amino groups by the treatment with (3-Aminopropyl)triethoxysilan (APTES, Sigma-Aldrich, St. Louis, MO, USA) (see Figure 1). These amino groups were used for conjugation of the fluorophore 5(6)-carboxyfluorescein N-hydroxysuccinimide ester (FITC, Sigma-Aldrich, St. Louis, MO, USA) and in a second reaction the EGFR-antibody (Figure 2). 

The attachment of the EGFR antibody [EGF receptor (D38B1) XP^®^ rabbit mAb, #4267, (EGFR-Ab) or EGF receptor (D38B1) XP^®^ rabbit mAb (Alexa Fluor^®^ 555 conjugate), #5108, (AF555-EGFR-Ab), both Cell Signaling Technology, Danvers, MA, USA] to the SiO_2_ particles is performed by an 1-ethyl-3-(3-dimethylaminopropyl)carbodiimide (EDC, Thermo Fisher Scientific, Rockford, IL, USA)-N-hydroxysuccinimide (NHS, Thermo Fisher Scientific, Rockford, IL, USA)-coupling using the terminal carboxy group of the antibodies and the primary amino groups located on the particles’ surface (Figure 2). The resulting amide bond links the antibody covalently to the particle. The EGFR-antibody is aimed at binding the EGF receptor overexpressed on the tumor cells to mark these cells with the FITC-conjugated SiO_2_-NPs. 

The preparation was conducted as follows: First, two aliquots of 3 mg FITC-SiO_2_-NPs were washed thrice with 0.9% sodium chloride (NaCl, Fresenius Kabi, Bad Homburg, Germany). The NPs were centrifuged (13,000 rpm, 10 min, room temperature (RT)), the supernatants were removed, and the NPs were dispersed by sonication and vortexing. In the next step, 2 mg/mL FITC-SiO_2_-NPs were incubated with NHS and EDC according to the manufacturer’s instructions. The EGFR-antibody was added to one aliquot (262.5 ng, EGFR-Ab or AF555-EGFR-Ab) and 15 µL 0.9% NaCl were added to the second aliquot (FITC-SiO_2_-NPs). Then, the coupling was performed for 2 h at 12 °C in a Thermomixer comfort (Thermo Fisher Scientific, Rockford, IL, USA) at 1400 rpm. The nanoparticles were centrifuged, washed thrice with 0.9% NaCl and dispersed at 6 mg/mL in 0.9% NaCl.

**Analytical characterization:** The SiO_2_-NPs were characterized by transmission electron microscopy (TEM) and ζ-potential measurements. TEM images were recorded using a Philips EM420 microscope (Philips, Amsterdam, Netherlands) with an acceleration voltage of 120 kV. Samples for TEM were prepared by dropping a diluted solution of SiO_2_-NPs in ethanol onto a carbon coated copper grid (Plano, Wetzlar; Germany). ζ-potential measurements were conducted with a Malvern Zetasizer Nano ZS (Malvern Panalytical, Kassel, Germany). The SiO_2_-NPs were dispersed in 1 mL of ethanol or water, filtered (Millex-GS syringe filter, pore size 0.22 µm, Merck Millipore, Billerica, MA, USA) and transferred to Malvern cuvettes (Disposable capillary cell DTS1061). 15 ζ-potential measurements were carried out twice at 25 °C to determine mean ζ-potential. 

**Antibody coupling efficiency:** Aliquots of AF555-EGFR-FITC-SiO_2_-NPs and FITC-SiO_2_-NPs were measured with a Fluoroskan Ascent Microplate Reader (Thermo Fisher Scientific, Rockford, IL, USA) after AF555-EGFR-Ab conjugation with 485/538 and 538/600 nm filter pairs. The fluorescence of AF555-EGFR-Ab was divided by the FITC fluorescence of the NPs to determine the coupling efficiency. The experiment was repeated three times and a paired t-test was performed.

**Human specimen:** The use of human tissue specimens is protected under and is in consent with prior decisions from the ethics committee of the university medical center Mainz. Patients gave informed consent according to national legal guidelines and agreed to scientific use of excess biological material retrieved during treatment.

**Cell lines and culture conditions:** The human hepatocarcinoma cell line HuH7 [14] was obtained from RIKEN BioResource Center. A human base of tongue squamous cell carcinoma cell line HNSCCUM-02T was established in our laboratory [15]. Both cell lines were cultured in Dulbecco’s modified Eagle’s medium: Nutrient mixture F12 (DMEM/F12, Gibco, Thermo Fisher Scientific, Rockford, IL, USA), supplemented with 5% bovine calf serum iron supplemented (FCS, VWR Life Science Seradigm, Radnor, PA, USA), and 2% penicillin-streptomycin (both Sigma-Aldrich, St. Louis, MO, USA) (cell culture medium) at 37 °C in a humidified incubator at 5% (*V*/*V*) CO_2_. Primary human fibroblasts were isolated from nasal concha originating from a conchotomy. The fibroblasts were cultured in DMEM/F12 with 10% FCS and stained with a monoclonal antibody to fibroblast surface protein-ascites (Acris Antibodies Inc., Herford, Germany) for verification. At a confluence of 85% to 90%, the cells were trypsinized with Trypsin-EDTA (T/E, Sigma-Aldrich, St. Louis, MO, USA) and were counted using a hemocytometer (Brand GmbH & Co KG, Wertheim Germany). Depending on the assay performed, different cell counts were seeded and treated.

**Nanoparticle preparation:** Stocks of EGFR-FITC-SiO_2_-NPs (1 or 2 mg/mL) were prepared in high-purity H_2_O (Cayman Chemical Company, Ann Arbor, MI, USA) or 0.9% NaCl. The NP dispersions were sonicated using a Sonorex Super RK 510 H (BANDELIN electronics, Berlin, Germany) for 5 min at 32 W amplitude, prior to each experiment. 

**Cellular viability:** The alamarBlue^®^ assay was performed to analyze the potential effects of EGFR-FITC-SiO_2_-NPs on the viability of HuH7 and HNSCCUM-02T cells. Per well, 15,000 cells were seeded in a black 96-well plate with clear bottom (Greiner Bio-One International AG, Kremsmünster, Austria) and cultivated overnight for adherence. The cells were treated with 50 or 100 µg/mL EGFR-FITC-SiO_2_-NPs and FITC-SiO_2_-NPs in cell culture medium and controls (0 µg/mL) were treated with 5% 0.9% NaCl in cell culture medium, respectively. For a quantification of the viability, the medium was replaced after 24 h incubation by a medium including alamarBlue^®^ (10% *V*/*V*, Thermo Fisher Scientific, Rockford, IL, USA). The cells were incubated for 3 h at 37 °C and then the fluorescence was measured (Fluoroskan Ascent Microplate Reader, Ex: 538 nm, Em: 600 nm, Thermo Fisher Scientific, Rockford, IL, USA). The media were removed, the cells briefly washed with PBS and lysed with lysis buffer [0.07 µM sodium dodecyl sulphate (SDS), 0.05 M tris(hydroxymethyl)aminomethane hydrochloride (Tris), 1.57 M glycerol in water]. After several freeze and thaw cycles the protein content of each sample was determined with DC™ Protein Assay (Bio-Rad, Munich, Germany). Metabolic activity data were normalized to protein content and were expressed as percentage of the respective controls. The experiment was repeated three times. 

**Confocal laser scanning microscopy of in vitro staining with AF555-EGFR-FITC-SiO_2_-NPs and FITC-SiO_2_-NPs**: To show the binding of the AF555-EGFR-FITC-SiO_2_-NPs to the surface of EGF receptor expressing tumor cells, HNSCCUM-02T cells were seeded onto μ-slides 8-well ibidiTreat (ibidi GmbH, Munich, Germany) at 5000 cells per well. After adherence of the cells, 100 µg/mL AF555-EGFR-FITC-SiO_2_-NPs and FITC-SiO_2_-NPs were added for 30 min, respectively. Then, samples were washed with PBS (3 × 5 min), fixed with 4% paraformaldehyde (PFA) in PBS for 15 min and washed with PBS (3 × 5 min). Samples were fixed 4 min with ice-cold acetone, washed with PBS (3 × 5 min) and incubated with 1% bovine serum albumin (BSA) in PBS for 30 min. Cells were stained with AlexaFluor™ 350 Phalloidin (1:40, Invitrogen by Thermo Fisher Scientific, Waltham, MA, USA) in 1% BSA in PBS for 45 min, washed with PBS (3 × 5 min), rinsed briefly with double-distilled water and embedded in mounting medium (Vectashield, Vector Laboratories, Burlingame, CA, USA). All slides were examined using a Leica DMi8 confocal laser scanning microscope (Leica Microsystems, Wetzlar, Germany) at 630× magnification. Unfortunately, the Phalloidin staining was very weak and therefore not depicted in the results.

**Confocal laser scanning microscopy of in vitro staining with AF555-EGFR-FITC-SiO_2_-NPs and AF555-EGFR-Ab:** The contrast enhancement capabilities of AF555-EGFR-FITC-SiO_2_-NPs were compared to AF555-EGFR-Ab in HNSCCUM-02T cells and primary human fibroblasts. The cells were seeded in µ-slides 8-well ibidiTreat, let to adhere overnight, and pre-incubated with 1% BSA in PBS for 30 min. Then, the cells were treated for 30 min with 100 µg/mL AF555-EGFR-FITC-SiO_2_-NPs or the corresponding amount of AF555-EGFR-Ab in cell culture medium, respectively. The cells were washed and fixed as mentioned above and finally, the samples were embedded in VECTASHIELD^®^ Hardset™ Antifade Mounting Medium with DAPI (Vectashield, Vector Laboratories, Burlingame, CA, USA). Images were obtained with Leica DMi8 confocal laser scanning microscope (Leica Microsystems, Wetzlar, Germany) at 630× magnification.

**EGFR protein expression in cell lysates and floor of mouth lysate:** HNSCCUM-02T cells, HuH7 cells, and human fibroblasts were lysed with RIPA lysis buffer [65 mM Tris, 154 mM NaCl (Carl Roth, Karlsruhe, Germany), 1% NP-40 (Hoffmann- La Roche AG, Basel, Switzerland), 0.025% sodium deoxycholate (Sigma-Aldrich, St. Louis, MO, USA), 1 mM EDTA (Carl Roth, Karlsruhe, Germany), pH 7.4 with protease inhibitor cocktail (Complete^®^, Hoffmann- La Roche AG, Basel, Switzerland) and phosphatase inhibitor cocktail (PhosSTOP, Hoffmann- La Roche AG, Basel, Switzerland)]. Samples were sonicated for 30 s, incubated on ice for 20 min and sonicated twice for 30 s. In the next step, the lysates were centrifuged (13,000 rpm, 20 min, 12 °C, BioFuge Fresco, Heraeus, Kendro Laboratory Products, Hanau, Germany), and the supernatants were transferred to new centrifuge tubes. The floor of mouth tissue was weighed and cut into small pieces, liquid nitrogen was added, and the tissue sample was further ground in a mortar. This was repeated two to three times. Then, the sample was lysed with RIPA lysis buffer (volume dependent on the amount of sample) and transferred to a centrifuge tube. The lysate was sonicated twice for 30 s (75%), incubated on ice for 20 min and sonicated twice again. Finally, the sample was centrifuged (13,000 rpm, 20 min, 12 °C, BioFuge Fresco, Heraeus) and transferred to a fresh centrifuge tube. The protein concentration was determined with DC™ Protein Assay (Bio-Rad, Munich, Germany) in accordance with the manufacturer’s instructions. Samples were subjected to discontinuous polyacrylamide gel electrophoresis. 20 µg proteins per sample were separated in a 7.5% stain-free polyacrylamide gel (prepared according to the instructions by Bio-Rad) with 10 mA for 2.5 h. The stain-free gel was activated, and protein bands were transferred to a methanol-activated PVDF membrane with Biometra tank blot (20 V, overnight). The blotting buffer contained 48 mM Tris, 39 mM Glycine (Carl Roth, Karlsruhe, Germany) and 20% (*V*/*V*) methanol (Honeywell Riedel- de Haën^®^, Seelze, Germany) in desalted water. The membrane was briefly washed in 20 mM Tris-buffered saline (137 mM NaCl) containing 0.1% Tween20 (TBS-T_20_) and all protein bands were detected at ChemiDoc (Bio-Rad, Munich, Germany). After blocking with 5% dry-milk in TBS-T_20_ for 1 h at room temperature, the membrane was washed with TBS-T_20_ (3 × 5 min) and incubated overnight at 4 °C with EGFR-Ab 1:1000 in 5% BSA in TBS-T_20_ under agitation. The next day, the membrane was washed with TBS-T_20_ (3 × 5 min) and incubated with anti-rabbit IgG, HRP-linked antibody (Cell Signaling Technologies) 1:5000 in 5% dry-milk in TBS-T_20_ for 1 h at RT. The protein bands were detected at ChemiDoc™ MP Imaging System (Bio-Rad) after 2 min incubation with Western Lightning^®^ Plus ECL (PerkinElmer, Waltham, MA, USA) reagent. Then, the membrane was stripped at RT: 30 min with 25 mM glycine pH 2.0 and 30 min with 1% (m/V) SDS in desalted water. For the detection of β-actin, the membrane was blocked with 5% dry-milk in TBS-T_20_ (1 h at RT), washed with TBS-T_20_ (3 × 5 min), incubated with anti-β-actin monoclonal antibody, clone AC-15 (Sigma-Aldrich, St. Louis, MO, USA) 1:10,000 in 5% BSA in TBS-T_20_ overnight at 4 °C, washed again thrice, incubated with anti-mouse IgG, HRP-linked antibody (Cell Signaling Technologies Inc, Danvers, MA, USA) 1:5000 in 5% dry-milk in TBS-T_20_ (1 h, RT) and visualized with Western Lightning^®^ Plus ECL reagent at ChemiDoc™MP Imaging System.

**3D cell culture:** 3D solid tumors were prepared with the cell line HuH7 and 5,000,000 cells were used for one tumor, which was applied to the chorioallantoic membrane (CAM) assay. First, the cells were transferred to a 1.5 mL centrifuge tube and generated a cellular pellet by centrifugation (1400 rpm, 10 min, 20 °C, Hettich Universal 16R, Hettich Zentrifugen, Tuttlingen, Germany). The supernatants were carefully removed, the cells were quickly suspended in 25 µL Matrigel^®^ Basement Membrane Matrix (Corning^®^, Corning Inc, NY, USA) and pipetted into six-well plates. After 30 min incubation in the incubator (37 °C, 5% CO_2_ (*V*/*V*)), the tumor model stiffed, medium was added to the wells and the 3D-cultures were incubated overnight until use in the CAM assay. 

**Figure 3:**
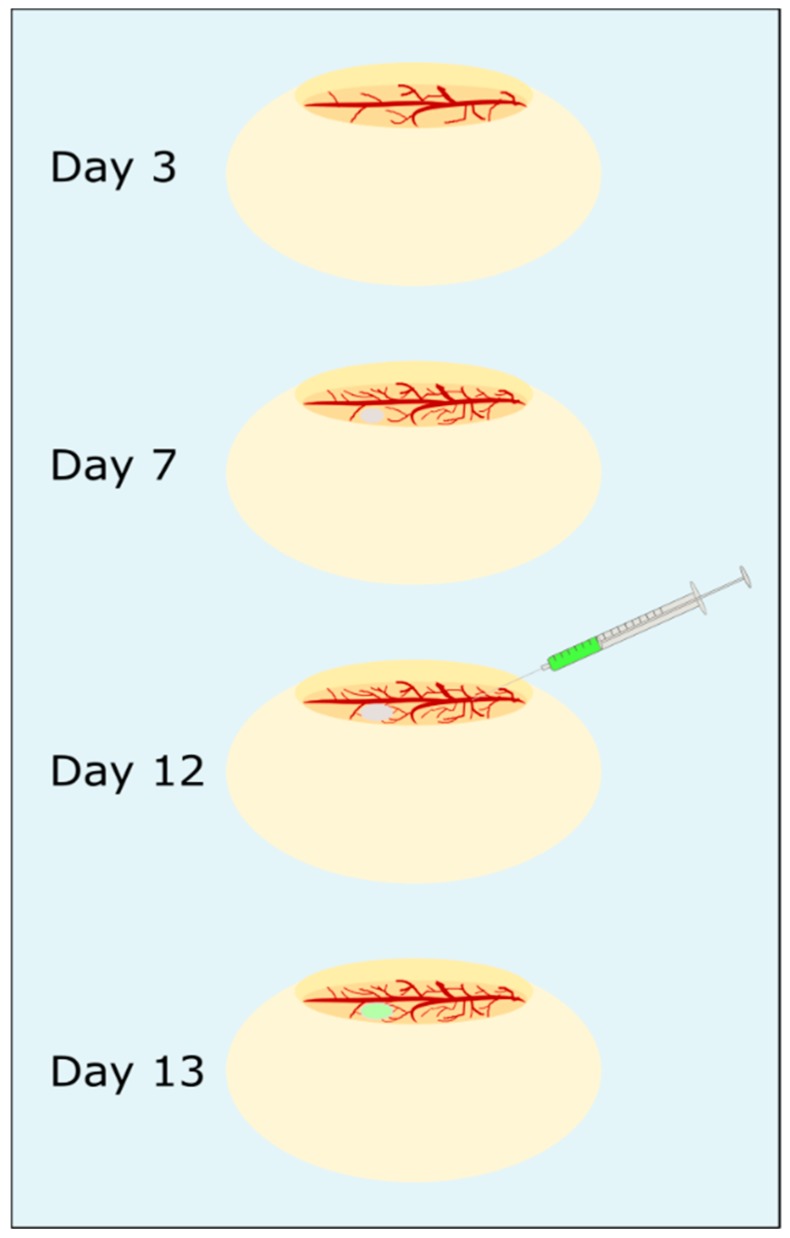
Chorioallantoic membrane assay as in vivo model. After three days incubation, fertilized chicken eggs were opened, and viability was assessed. On day 5, a 3D tumor of hepatocarcinoma cells was placed on the CAM and could grow for 5 days. Then, EGFR-FITC-SiO_2_-NPs or FICT-SiO_2_-NPs were injected in a blood vessel, respectively. The next day, the CAM with tumor and the liver were removed and prepared for confocal laser scanning microscopy.

**Chorioallantoic membrane assay:** The steps of the CAM assay are presented in Figure 3. Fertilized white leghorn eggs (LSL, Dieburg, Germany) were cleaned with sterilized water, screened for any damage and intact eggs were horizontally placed in a freshly cleaned incubator at 37.5 °C. After three days incubation, about 6 mL albumen were removed from each egg with a syringe and the hole was sealed with sticky tape. Then, a micropore bandage was attached horizontally to the top of the egg to prevent eggshells falling into the egg while an oval hole was cut out. The egg was checked for fertilization, sealed with Parafilm “M”™, and was further incubated. The chicken embryos (CEs) were monitored daily for viability. Viability was characterized by steady blood flow and visible heartbeat. On the seventh day of incubation, a blood vessel was carefully cut with a scalpel, a 3D-culture of HuH7 cells (preparation described above) was directly placed on the wound and 20 µL Matrigel^®^ Basement Membrane Matrix (Corning^®^, Corning Inc, Corning, NY, USA) were added to the cells. Then, the CEs were incubated further with minimal movement to enhance tumor growth. After the tumor grew for five days, 50 µL of 0.9% (m/V) NaCl solution, 1.0 mg/mL EGFR-FITC-SiO_2_-NPs in 20% ethanol/80% 0.9% NaCl (*V*/*V*) or FITC-SiO_2_-NPs in 0.9% NaCl were injected into a blood vessel and the wound was sealed immediately with a silver nitrate stick. The CEs were incubated further for 24 h and from two embryos of each treatment group the CAM with tumor and liver were removed, transferred to immunohistochemistry cassettes and fixed in 4% PFA overnight. Tissue samples were stored in 1% PFA until further processing. 

**Fluorescence staining of chicken embryonic tissues:** The washing and dehydration of the tissues were performed at RT and under light protection. First, the fixed tissues were washed three times with deionized water (20 min each) and then incubated in 70%, 80% and 90% (*V*/*V*) isopropanol (Hedinger, Stuttgart, Germany) in deionized water for one hour each. Next, the samples were incubated in 100% isopropanol for 1.5 h, afterwards for two hours in xylene (PanReac AppliChem, Darmstadt, Germany) and finally overnight in fresh xylene. The cassettes were transferred to liquid paraffin, incubated for four hours at 60 °C, transferred to freshly prepared liquid paraffin and incubated overnight at 60 °C. The embedding of the tissues was conducted with Leica EG 1140H tissue embedding center. The organs were placed in a small form, liquid paraffin was added, and the form was placed on a cooling plate. Paraffin tissue sections were cut with a Leica RM2165 rotary microtome to 3–5 µm sections and placed on microscope slides. Next, the tissue sections were deparaffinated as follows: Incubation in xylene for 5 min (twice), incubation in 100%, 90%, 80%, 70% (*V*/*V*) isopropanol in desalted water for 5 min each and 5 min of incubation in deionized water. Finally, the samples were embedded in Vectashield^®^ Hardset™ Antifade Mounting Medium with DAPI (Vectashield) and solidified for at least 24 h before microscopic analysis. The stained samples were analyzed with a Leica DMi8 confocal laser scanning microscope with 400-fold magnification.

**Immunohistochemistry staining of HNSCCUM-02T cells and HuH7 cells:** HNSCCUM-02T and HuH7 cells were detached with trypsin/EDTA, transferred to 1.5 mL centrifuge tubes, centrifuged (350 g, 5 min, RT) and washed with PBS three times. The PBS was removed completely after the final washing step, and 4% PFA in PBS were added. The samples were stored at 4 °C. Then, cells were washed three times with deionized water and dehydrated in 70%, 80%, 90% and 100% (*V*/*V*) isopropanol in deionized water for 30 min each. Next, the cellular pellets were incubated twice in xylene and twice in liquid paraffin for 30 min each. The cell pellets were generated by centrifugation (4000 rpm, 5 min, RT, Universal 16 R, Hettich Zentrifugen, Tuttlingen, Germany) after each incubation step. After the final paraffin incubation step, the paraffin containing cell pellet was solidified briefly, transferred carefully to an immunohistochemistry cassette and embedded in paraffin with a Leica EG 1140H tissue embedding center (Leica Biosystems, Wetzlar, Germany). The samples were cut to 3–5 µm paraffin sections with a Leica RM2165 rotary microtome (Leica Biosystems, Wetzlar, Germany) and placed on microscope slides. Then, cellular samples were deparaffinated as described above, washed once with TBS-T_20_ (5 min) and antigen demasking was performed with 10 mM Tris/1 mM EDTA (pH 9.00) in desalted water for 30 min in a steamer (Steam Cuisine High Speed). After rinsing the sample briefly with deionized water, it was washed for 5 min with TBS-T_20_, blocked for 45 min with goat serum (normal) (DAKO Agilent, Santa Clara, CA, USA) 1:10 in 1% BSA in PBS and was incubated with EGFR-Ab 1:50 in 1% BSA in PBS for one hour at RT. The samples were washed twice with TBS-T_20_ for 5 min each, incubated with DEnVision™+Dual Link System-HRP (DAKO Agilent, Santa Clara, CA, USA) for 30 min and washed again twice with TBS-T_20_ for 5 min. For visualization the samples were incubated with DakoCytomation Liquid DAB+ Substrate Chromogen System (DAKO Agilent, Santa Clara, CA, USA) and hemalum solution acid according to Mayer ready-to-use (Carl Roth, Karlsruhe, Germany) for 5 min each and washed for 5 min with tap water. Finally, the samples were dehydrated by incubating in 70%, 80%, 90% and 100% isopropanol in desalted water and twice with xylene for 5 min each and embedded in Eukitt^®^ Quick-hardening mounting medium (Sigma-Aldrich, St. Louis, MO, USA). Images were taken at a Nikon Eclipse E200 microscope with 400-fold magnification.

**Confocal endomicroscopy:** Tumor samples (oropharynxcarcinoma) and healthy tissue (gingiva samples) were incubated briefly in 100 µg/mL EGFR-FITC-SiO_2_-NP or FITC-SiO_2_-NP dispersions, respectively, and rinsed with deionized water. A miniaturized confocal microscope was integrated into the distal tip of a conventional video endoscope with an outer diameter of 13.2 mm (Pentax, Tokyo, Japan, EC 3830FK) and used for visualization. A single optical fiber was operated as the illumination point source and the detection pinhole. A solid-state laser delivered an excitation wavelength of 488 nm at a maximum laser power output of 1 mW or lower at the tissue surface. Confocal image data were collected with 1100-fold magnification at a scan rate of 0.8 frames/s (1024 × 1024 pixels) and an image size of 215.9 × 215.9 µm [1].

**Fluorescence staining of tumor and healthy tissue samples:** After confocal endomicroscopic analysis, the tumor samples and healthy mucosa (gingiva) were fixed in 4% PFA. The samples were dehydrated and embedded in paraffin as described above. Paraffin sections with a thickness of 3–5 µm were prepared and deparaffinated as mentioned before. Next, the samples were embedded in Vectashield^®^ Hardset™ Antifade Mounting Medium with DAPI and solidified for at least 24 h before microscopic analysis with an Eclipse TE2000-U fluorescence microscope (Nikon GmbH, Dusseldorf, Germany). 

## 3. Results

**Figure 4:**
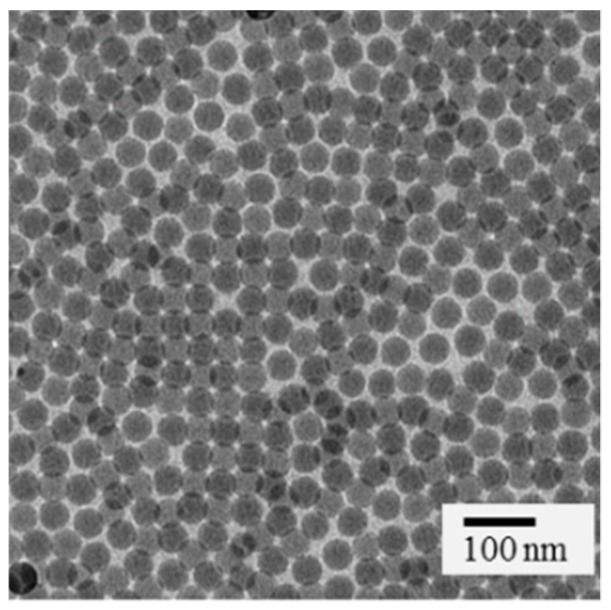
TEM image of 45 nm SiO_2_-NPs. SiO_2_-NPs exhibit a uniform and spherical shape.

The spherical SiO_2_-NPs exhibited a mean diameter of 45 nm and a mean ζ-potential of −8 ± 2 mV. After functionalization with primary amino groups, the ζ-potential increased to 10 ± 2 mV, confirming successful functionalization. Nanoparticles could easily be dispersed before and after antibody conjugation, and they showed a uniform size and morphology as shown in Figure 4. Thus, EGFR-FITC-SiO_2_-NPs are suited as a nano-contrast agent.

**Figure 5:**
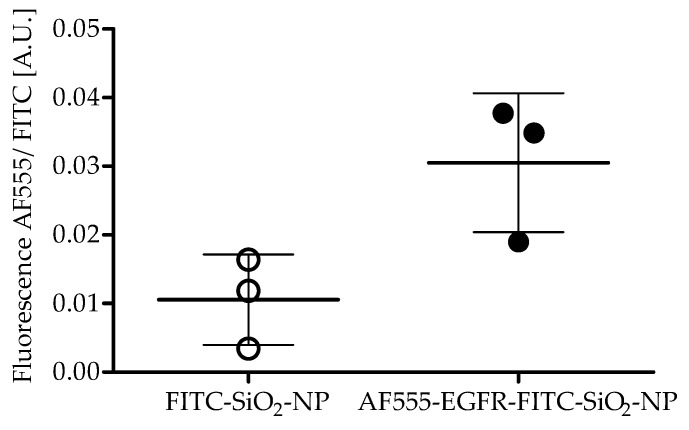
Coupling efficiency of Alexa Fluor 555 labeled EGFR-antibody to FITC-SiO_2_-NPs. The quotient of AF555 and FITC fluorescence was significantly greater (2.9-fold) for AF555-EGFR-FITC-SiO_2_-NPs than for FITC-SiO_2_-NPs. Mean ± S.D., paired t-Test, P = 0.0230, n = 3.

The antibody coupling efficiency was determined with an Alexa Fluor^®^ 555-labeled EGFR-antibody (AF555-EGFR-Ab). The fluorescence of Alexa Fluor^®^ 555 after antibody conjugation was normalized to the fluorescence of FITC to evaluate the coupling efficiency. The quotient of the fluorescence of AF555 and FITC was significantly greater (2.9-fold) for AF555-EGFR-FITC-SiO_2_-NPs than for FITC-SiO_2_-NPs, indicating a successful antibody conjugation, as shown in Figure 5.

**Figure 6:**
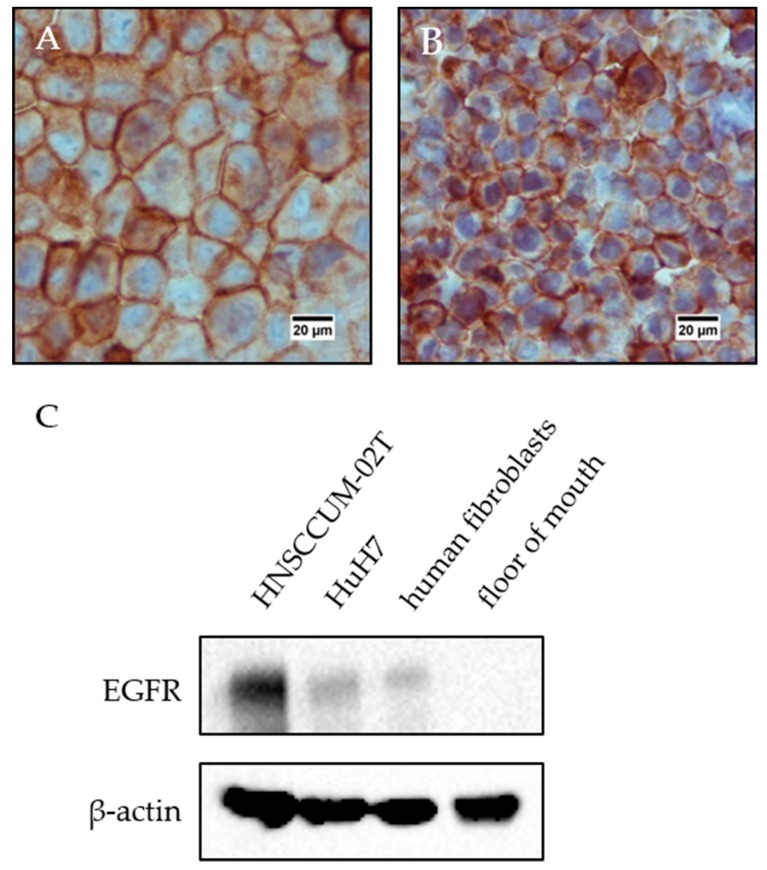
EGFR expression in HNSCCUM-02T and HuH7 cells. (**A**) HNSCCUM-02T cells and (**B**) HuH7 cells highly express EGFR. (**C**) Immunoblot analysis revealed high EGFR expression in HNSCCUM-02T cells and weak EGFR expression in HuH7 cells and primary human fibroblasts as well. In contrast, no EGFR expression was detected in a floor of mouth sample.

The cell lines were analyzed for EGFR expression by immunohistochemistry and protein expression analysis (Figure 6). Both cell lines expressed EGFR. Therefore, both cell lines are suited for EGFR-targeting experiments. Additionally, non-malignant human primary fibroblasts exhibited a weak EGFR expression and no EGFR expression was detected in healthy floor of mouth tissue.

**Figure 7:**
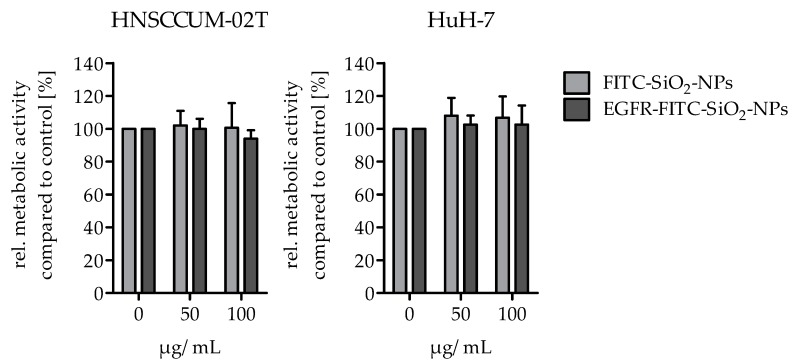
Biocompatibility of FITC-SiO_2_-NPs and EGFR-FITC-SiO_2_-NPs. 50 µg/mL and 100 µg/mL FITC-SiO_2_-NPs and EGFR-FITC-SiO_2_-NPs did not reduce the viability of HNSCCUM-02T and HuH7 cell lines after 24 h incubation, respectively. Mean + S.D., n.s., n = 3.

Next, we evaluated the cellular toxicity of the synthesized nano-contrast agent in HNSCCUM-02T and HuH-7 cells. EGFR-FITC-SiO_2_-NPs and FITC-SiO_2_-NPs did not reduce the viability as a measure for cellular toxicity at 50 µg/mL and 100 µg/mL nanoparticles (Figure 7). Hence, the EGFR-FITC-SiO_2_-NPs exhibited a good biocompatibility even after 24 h of incubation.

**Figure 8:**
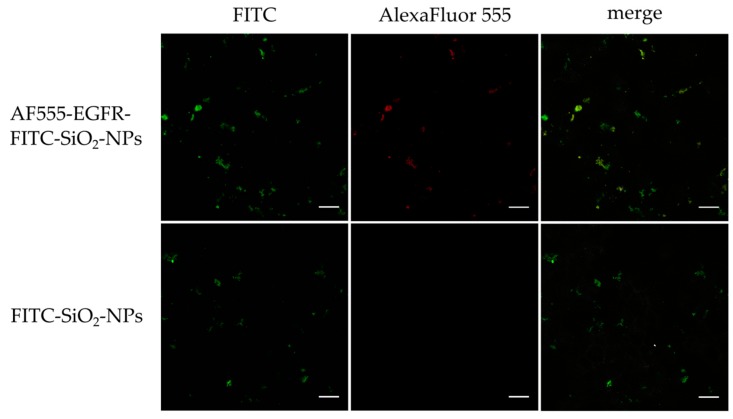
Cellular binding of AF555-EGFR-FITC-SiO_2_-NPs and FITC-SiO_2_-NPs in vitro. HNSCCUM-02T cells were incubated with AF555-EGFR-FITC-SiO_2_-NPs and FITC-SiO_2_-NPs for 30 min. Samples were fixed and analyzed with confocal laser scanning microscopy. Signal overlap of FITC (green) and Alexa Fluor^®^ 555 (red) is presented in yellow. Representative images are shown. Co-localization of AF555 with FITC-labeled SiO_2_-NPs was observed for AF555-EGFR-FITC-SiO_2_-NPs. Slightly more AF555-EGFR-FITC-SiO_2_-NPs bound to HNSCCUM-02T cells than FITC-SiO_2_-NPs. Scale: 20 µm.

The cell line HNSCCUM-02T was incubated with AF555-EGFR-FITC-SiO_2_-NPs and FITC-SiO_2_-NPs for 30 min to evaluate nanoparticle binding in vitro. AF555-EGFR-Ab signal and FITC signal were observed with confocal laser scanning microscopy and binding of nanoparticles to the cellular membranes was noted. The presence of AF555-EGFR-Ab concurrent with FITC on FITC-labeled silica nanoparticles was indicated by signal overlap, which is depicted in yellow and indicated successful antibody conjugation. No AF555 signal was detected for FITC-SiO_2_-NPs. Slightly more EGFR-FITC-SiO_2_-NPs than FITC-SiO_2_-NPs were bound to the cells as presented in Figure 8.

**Figure 9:**
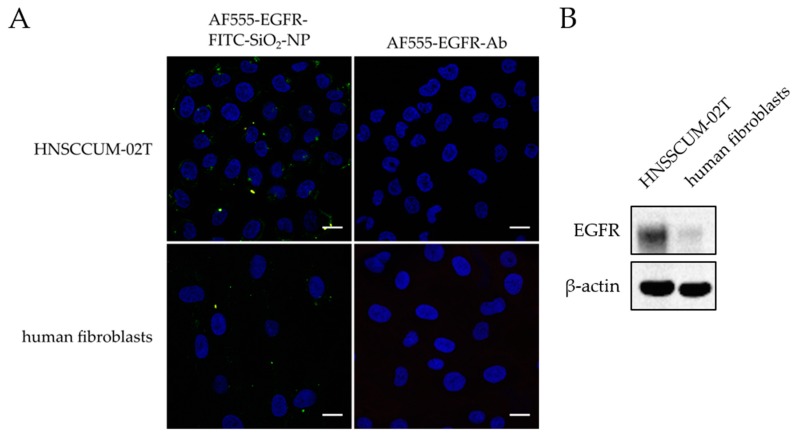
Comparison of AF555-EGFR-FITC-SiO_2_-NPs and AF555-EGFR-Ab immunostaining in HNSCCUM-02T and human fibroblasts. (**A**) HNSCCUM-02T cells and primary human fibroblasts were incubated with 100 µg/mL AF555-EGFR-FITC-SiO_2_-NPs or the corresponding amount of AF555-EGFR-Ab for 30 min at 37 °C. Then, the cells were washed, fixed, and embedded in DAPI-containing mounting medium. The nuclei are shown in blue, the FITC-SiO_2_-NPs in green, the AF555-EGFR-Ab in red, and the co-localization in yellow. More AF555-EGFR-FITC-SiO_2_-NPs attached to the cellular membranes of HNSCCUM-02T cells than primary human fibroblasts. The AF555-EGFR-Ab alone did not stain the cells. Thus, FITC-SiO_2_-NPs are required for contrast enhancement and antibody-conjugation improved the specificity. Scale: 20 µm. (**B**) Immunoblot analysis of EGFR expression in HNSCCUM-02T cells and primary human fibroblasts. HNSCCUM-02T cells express much more EGFR than the used primary human fibroblasts. β-Actin was used as the loading control.

Furthermore, we compared the contrast enhancement capability of the nanoparticulate contrast agent with an immunostaining in HNSCCUM-02T cells and primary human fibroblasts (Figure 9A). Only when the AF555-EGFR-Ab was conjugated to the FITC-SiO_2_-NPs, the cellular membranes were marked, and a co-localization of the AF555-EGFR-Ab and the FITC-SiO_2_-NPs was noticed. More AF555-EGFR-FITC-SiO_2_-NPs bound to HNSCCUM-02T cells than primary human fibroblasts. This is consistent with the EGFR expression of these cells, as shown in Figure 9B. The used primary human fibroblasts exhibited only a weak EGFR expression while HNSCCUM-02T cells had a high EGFR expression.

**Figure 10:**
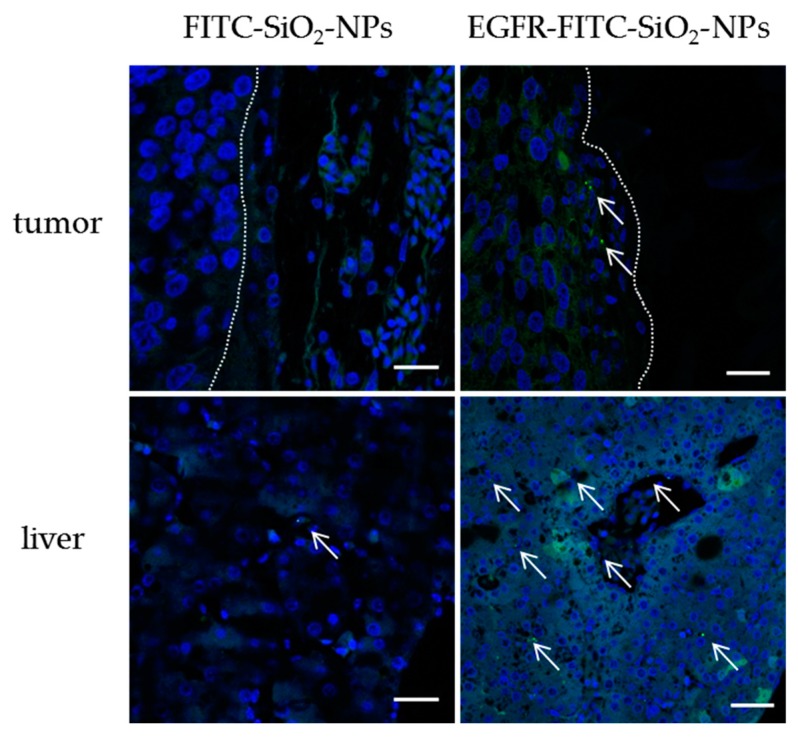
FITC-SiO_2_-NPs and EGFR-FITC-SiO_2_-NPs in vivo distribution. 50 µL of 1 mg/mL FITC-SiO_2_-NPs in 0.9% NaCl or EGFR-FITC-SiO_2_-NPs in 20% ethanol/80% 0.9% NaCl were injected into the CAM and chicken embryos were incubated for 24 h, respectively. The CAM with tumor and the liver were removed, fixed and processed for paraffin sectioning. The paraffin sections were stained with DAPI. Nuclei are shown in blue and FITC-labeled nanoparticles in green (arrows). A dotted line indicates the tumor border; the tumor is on the left. EGFR-FITC-SiO_2_-NPs were found occasionally in the tumor and often in the liver. FITC-SiO_2_-NPs could be rarely detected in the liver. Scale: 20 µm.

Yet, EGFR-FITC-SiO_2_-NPs and FITC-SiO_2_-NPs showed a great variance in binding to head and neck cancer cells in cell culture. For that reason, we applied EGFR-FITC-SiO_2_-NPs and FITC-SiO_2_-NPs to an in vivo model. A tumor consisting of HuH7 cells was established on a chicken embryo’s chorioallantoic membrane (CAM). HuH7 cells were used because the cells formed more reliable tumors than HNSCCUM-02T cells on the CAM. EGFR-FITC-SiO_2_-NPs and FITC-SiO_2_-NPs were administered into a blood vessel and after 24 h the CAM with tumor and the liver were removed for microscopic analysis. We found that ethanol enhanced the fluorescence of the nanoparticles and therefore injected EGFR-FITC-SiO_2_-NPs in ethanol/NaCl (1:4). Paraffin sections were prepared and stained with DAPI. The observations are shown in Figure 10 by representative images. EGFR-FITC-SiO_2_-NPs were occasionally found in the tumor, whereas no FITC-SiO_2_-NPs were found in the CAM or the tumor. However, EGFR-FITC-SiO_2_-NPs frequently occurred in the liver, the organ with the highest blood circulation. FITC-SiO_2_-NPs were rarely detected in the liver. Hence, targeted silica nanoparticles were still detected in an in vivo application after a long circulation time (24 h) but accumulated in the liver.

**Figure 11:**
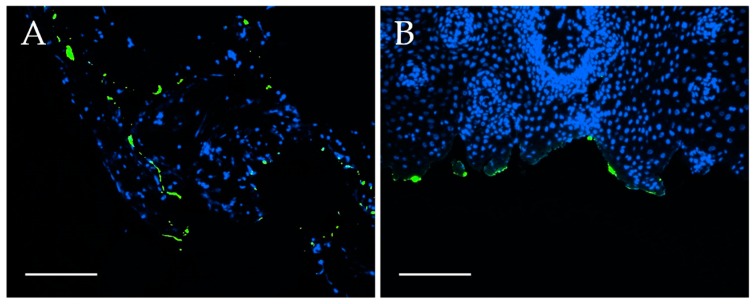
EGFR-FITC-SiO_2_-NP binding ex vivo. Fluorescence images of (**A**) oropharynx carcinoma and (**B**) healthy gingiva samples after incubation with EGFR-FITC-SiO_2_-NPs for two minutes. EGFR-FITC-SiO_2_-NPs are depicted in green, nuclei in blue. EGFR-FITC-SiO_2_-NPs attached preferably to tumor tissue while only few EGFR-FITC-SiO_2_-NPs bound to healthy tissue. Representative images are shown. Scale: 50 µm.

Next, freshly explanted tumor samples and healthy tissue samples were briefly incubated with EGFR-FITC-SiO_2_-NPs in vitro, fixed, processed for paraffin sectioning and stained with DAPI. More EGFR-FITC-SiO_2_-NPs attached to tumor tissue than healthy tissue as shown in Figure 11 in fluorescence images. 

**Figure 12:**
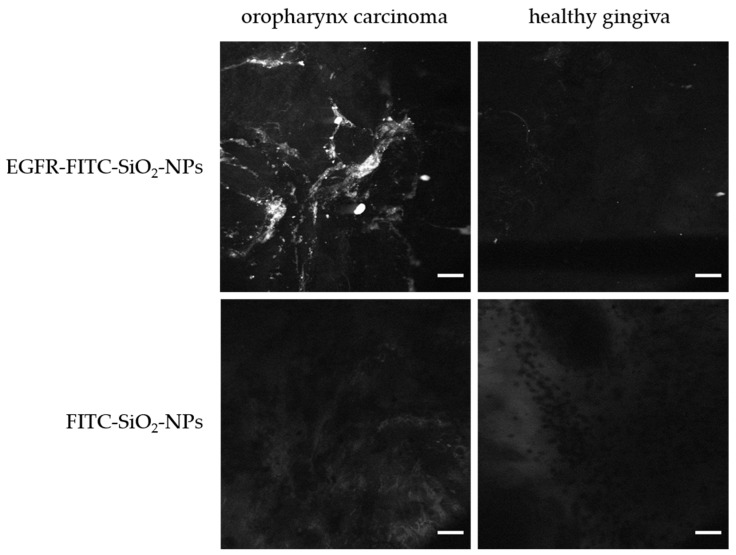
Confocal laser endoscope imaging of oropharynx carcinoma and healthy gingiva. Tissue samples were briefly incubated with EGFR-FITC-SiO_2_-NPs and FITC-SiO_2_-NPs, respectively, rinsed and observed with a confocal laser endomicroscope at 1100-fold magnification. EGFR-FITC-SiO_2_-NPs exhibited a bright signal in oropharynx carcinoma. Only background signal was detected in healthy gingiva and for FITC-SiO_2_-NPs. Scale: 25 µm.

Furthermore, oropharynxcarcinoma and healthy gingiva were incubated in 100 µg/mL FITC-SiO_2_-NP or EGFR-FITC-SiO_2_-NP dispersions for two minutes, respectively and observed with a confocal laser endomicroscope under conditions according to a usual endomircoscopic procedure. Representative images are displayed in Figure 12 in greyscale, which corresponds to the display the surgeon can observe. Only the tumor sample showed a signal of EGFR-FITC-SiO_2_-NPs while the FITC-SiO_2_-NPs (controls) exhibited no signal. Therefore, EGFR-FITC-SiO_2_-NPs preferably bind to tumor tissue, and the malignant area can be visualized by confocal laser endomicroscopy.

## 4. Discussion

In this study, we describe a novel approach for intraoperative real-time tumor detection. To the best of our knowledge this is the first report using anti-Epidermal Growth Factor Receptor (EGFR) antibodies attached to fluorescence-tagged silica nanoparticles for contrast enhancement. This nanoparticulate agent is highly specific, resulting in local contrast enhancement. Our study suggests a diagnostic approach of modified silica nanoparticles in combination with real-time endomicroscopy for in vivo tumor border evaluation.

The nanoparticles were synthesized by a novel method with short reaction times and controllable size and morphology. Additionally, simple amino functionalization of the particles was possible to allow fluorophore and EGFR-antibody binding. The three main components (nanoparticles, fluorophore, targeting moiety) can be replaced by others according to the respective goal. This nano-contrast agent is stable in acidic and basic environments and non-toxic as supported by biocompatibility analysis. Still, the amount of EGFR-Ab that was conjugated to the FITC-SiO_2_-NPs was relatively small and could not be quantified exactly. The synthesis could be modified to enhance EGFR-Ab coupling e.g., by using more antibody or by conducting the FITC and antibody conjugation simultaneously. Besides, the nanoparticle dispersion can easily be applied topically to the suspicious tissue to enhance the contrast of the tumor border and to allow easier surgical intervention. We favor topical application because it is less invasive than intravenous application, less EGFR-FITC-SiO_2_-NPs would be needed, and the risk of systemic adverse effects would be reduced. Furthermore, the silica nanoparticles bind to expressed EGFR of the tumor cells in a short time. Therefore, the surgeon can quickly distinguish malignant and normal tissue after excitation of the fluorescence dye and perform an optimal R0 resection. Before, we intravenously administered fluorescein to visualize the blood vessels, red blood cells, surface epithelial cells and the connective tissue matrix of the lamina propria. However, a clear discrimination between healthy and malignant tissue was not possible [6]. The use of EGFR-targeted and FITC-conjugated nanoparticles allows for a much more effective evaluation of suspicious lesions by identifying EGFR overexpressing areas.

In vitro, we did not find a significant difference in nanoparticle binding to EGFR overexpressing head and neck cancer cells between EGFR-targeted and untargeted FITC-SiO_2_-NPs. However, more AF555-EGFR-FITC-SiO_2_-NPs than FITC-SiO_2_-NPs seemed to bind to the cells after 30 min of incubation and AF555 signal overlap with FITC indicated successful conjugation of the AF555-EGFR-Ab to the FITC-SiO_2_-NPs. Additionally, more AF555-EGFR-FITC-SiO_2_-NPs were associated with HNSCCUM-02T cells than primary human fibroblasts and the AF555-EGFR-Ab alone did not stain the cells. Moreover, EGFR-FITC-SiO_2_-NPs were detected in the tumor and the liver in the CAM assay while FITC-SiO_2_-NPs were only rarely detected in the liver. Compared to the confocal endomicroscopy data, which indicated no FITC-SiO_2_-NP binding to malignant tissue, the FITC-SiO_2_-NP binding to cancer cells was high. This could have been caused by the proteins present in the cell culture medium, which formed a protein corona around the nanoparticles and reduced the targeting effect of the conjugated EGFR-antibody [16]. Lesniak et al. found that silica nanoparticles (diameter 50 nm) with a protein corona had much lower cellular binding and uptake than nanoparticles without a protein corona [17]. Here, the tissue samples were incubated with nanoparticles in PBS—hence, no protein corona could be formed. Therefore, EGFR-targeting of the silica nanoparticles had a greater effect in a short incubation time on binding to tissue samples than to cells where a protein corona was presumably present on both particle types. 

The use of EGFR-targeted silica nanoparticles was applied by Wan et al. as well to detect lung cancer in mice [18]. They used the near-infrared dye methylene blue as contrast agent and encapsulated it in silica nanoparticles to enhance the stability of the organic dye in vivo. In vivo tumor imaging in mice indicated an increase in methylene blue signal over time and a higher intensity for EGFR-targeted nanoparticles compared to untargeted nanoparticles [18]. Yet, methylene blue is a monoamine oxidase inhibitor and can cause serotonin syndrome when applied together with serotonergic drugs [19,20]. Therefore, clinical translation is difficult.

A different approach to detect small tumors by fluorescent labeling was described by Ueo et al. They employed an aminopeptidase-targeted activatable fluorophore probe for detection of malignant lesions in breast tissue within 5 to 15 min after application [21,22].

Still, a great advantage is the topical applicability of the silica-based contrast agent. Nevertheless, systemic application seems feasible because of the low toxicity profile of the particles suggesting this novel type of nanoparticles as a prototype for the future synthesis of drug-like particles for the use in medical or diagnostic purposes in human. We here used the CAM assay to analyze in vivo distribution of the nanoparticles and could identify EGFR-FITC-SiO_2_-NPs in the tumor and in the liver. This is not surprising, considering the liver is the organ with the highest blood flow. However, systemic delivery would need much higher particle amounts than topical application and more safety and tolerability studies would be needed. Additionally, our aim is a local contrast enhancement for a short time. Therefore, the topical and thereby local application is more reasonable.

## 5. Conclusions

In this proof-of-principle study we evaluated EGFR-targeted and FITC-labeled SiO_2_-NPs as contrast enhancer for confocal laser endomicroscopy. The EGFR-FITC-SiO_2_-NPs showed good biocompatibility, a successful binding of the EGFR-antibody was observed, and the EGFR-FITC-SiO_2_-NPs were detected in vitro, in vivo and ex vivo after short incubation times. Therefore, we are confident that this nano-contrast agent can improve the real-time in vivo analysis of suspicious mucosa and enables operative tumor border examination by confocal laser endomicroscopy. Overall, it has the potential to improve intraoperative definition of tumor free resection margins for head and neck squamous cell carcinoma by contrast enhancement.
